# Supplementary material for: The past, current, and future of neonatal intensive care units with artificial intelligence: a systematic review
Source: NPJ Digit Med. 2023 Nov 27;6:220. doi: 10.1038/s41746-023-00941-5 (PMC10682088; doi:10.1038/s41746-023-00941-5)
Supplement: Supplementary file 1 — Supplementary information [file 41746_2023_941_MOESM1_ESM.docx]

**Supplementary information**

**Full Search Strategy and Bias Analysis**

We used PubMed™, IEEEXplore™, Google Scholar™, and ScienceDirect™ to search for publications relating to AI, ML, and DL applications towards neonatology. We have done a varying combination of the keywords( i.e., one from technical keywords and one from clinical keywords) for the search. Clinical keywords were “infant,” “neonate,” “prematurity,” “preterm infant,” “hypoxic ischemic encephalopathy,” “neonatology,” “intraventricular hemorrhage,” “infant brain segmentation,” “NICU mortality,” “infant morbidity,” “ bronchopulmonary dysplasia,” “retinopathy of prematurity.” The inclusion criteria were (i) publication date between 1996-2022 and, (ii) being an artificial intelligence in neonatology study, (iii) written in English, (iv) published in a scholarly peer-reviewed

journal, and (v) conducted an assessment of AI applications in neonatology objectively. Technical keywords were AI, DL, ML, and CNN. Review papers, commentaries, letters to the editor and papers with only technical improvement without any clinical background, animal studies, and papers that used statistical models like linear regression, studies written in any language other than English, dissertation thesis, posters, biomarker prediction studies, simulation-based studies, studies with infants are older than 28 days of life, perinatal death, and obstetric care studies were excluded. An electronic reference manager (EndNote version 20) was utilized for reference organization. The article selection process involved two authors who independently performed the selection in two distinct phases, preceded by a pilot training test. In the initial phase, an assessment of titles and abstracts was carried out, alongside the application of predefined eligibility criteria. Subsequently, during the second phase, a thorough examination of full-text articles was undertaken by the reviewers, consistently aligning with the predetermined eligibility standards. Instances of variance were resolved through mutual agreement between the two authors. Following the first literature searches, each study's title and abstract were examined, and subsequently, studies that appeared to be possibly relevant were further evaluated for eligibility. The PRISMA flow diagram (Figure 2) contains comprehensive details regarding the study selection procedure. The preliminary investigation yielded a substantial collection of articles, amounting to approximately 9000 in total. To ensure accuracy and pertinence, we implemented a systematic and methodical procedure to carefully evaluate and choose publications that closely corresponded to our research objectives, study methodology, and the topic under investigation by following PRISMA 2020 guidelines^58^. Through a meticulous examination of the abstracts of the papers, a subset of 987 research was found (Figure 2). Ultimately, 106 studies were selected for inclusion in our systematic review. The evaluation encompassed diverse aspects, including sample size, methodology, data type, evaluation metrics, advantages, and limitations of the studies (Tables 2-7).

The included articles were assessed by both authors independently using the revised Cochrane risk-of-bias tool for non-randomized studies and were categorized into low risk, some concerns, or high risk. The risk of bias in the included studies was further evaluated using the QUADAS-2 (Quality Assessment of Diagnostic Accuracy Studies 2) tool^59-61^. The formal investigation of heterogeneity using meta-analysis was not possible due to the limited data availability. Additionally, the review protocol was not registered due to the same restriction.
